# Assessment of Nanoscale Configuration of Dentin in Non-carious Cervical Lesions

**DOI:** 10.7759/cureus.48003

**Published:** 2023-10-30

**Authors:** Akshat Sakkaria, Ramya Ramadoss, Nitya Krishnasamy, Sandhya Sundar, Suganya Panneerselvam, Pratibha Ramani

**Affiliations:** 1 Oral Biology, Saveetha Dental College and Hospitals, Chennai, IND; 2 Oral and Maxillofacial Pathology, Saveetha Dental College and Hospitals, Chennai, IND

**Keywords:** non-carious cervical lesion, dentinal tubules, eds, sem, dentin hypersensitivity

## Abstract

Introduction

Non-carious cervical lesions (NCCLs) are structural deformities or tissue loss in the cervical region of teeth, which are closer to the gum line that do not result from caries (tooth decay). On the tooth enamel at the gingival margin, these lesions are frequently seen as V- or U-shaped notches. The causes of NCCLs are multifaceted and involve intricate relationships between chemical, mechanical, and biological elements. The slow loss of enamel and dentin in the cervical area occurs due to mechanical causes like abrasive toothbrushing and occlusal tension, as well as chemical variables such as erosive acidic food components. As NCCLs advance, dental discomfort, aesthetic issues, and impaired tooth function may result. The aim of this study was to assess the nanoscale configuration of dentin in non-carious cervical lesions using scanning electron microscopy (SEM) and energy-dispersive spectroscopy (EDS).

Methodology

Sterilized teeth samples were selected from the extracted tooth repository of Saveetha Dental College and Hospitals, Chennai. Tooth samples were thinly sliced using a diamond bur. The sliced teeth were examined using a scanning electron microscope. Ionic configuration was assessed using EDS and elemental analysis.

Results

The findings showed the nanoscale morphology and elemental configurations present. Elemental mapping showed specific elemental localization in the affected area.

Conclusion

NCCLs can be attributed as a predominant factor leading to gingival recession causing dentin exposure and hypersensitivity. NCCLs can also threaten the integrity of dentin; therefore, early diagnosis of non-carious cervical lesions is crucial to ensure proper treatment plan and therapeutic regimens.

## Introduction

Non-carious cervical lesions (NCCLs) refer to tooth decay that occurs on surfaces of the teeth other than the cervical (neck) region. This can include decay on the biting surfaces (occlusal surfaces) of molars and premolars, as well as decay on the smooth surfaces (mesial, distal, buccal, and lingual) of any tooth in the mouth [1]. Due to the tooth's unique position and function, the vestibular side is where it occurs most frequently. As these lesions increase in frequency, they produce a number of unpleasant symptoms and point to more severe issues with the stomatogastric system. They are getting increasingly significant as human life expectancy is rising and oral health's significance is becoming more widely recognised [2]. Non-carious processes account for up to 25% of the pathological deterioration of the hard tooth tissue. These types of caries lesions are caused by various factors, including poor oral hygiene, a diet rich in sugars and carbohydrates, lack of fluoride, abfraction, abrasion and erosion. Biting forces can cause stress concentration, bruxism, premature contacts, occlusal interferences and clenching could also be contributory factors in the etiology of NCCLs [3]. Treatment for non-cervical caries lesions typically involves removing the affected portion of the tooth and treating the cavity with a restorative material, such as amalgam or composite resin. It is important to address these lesions promptly to prevent further decay and potential tooth loss. The lesions might appear as minor depressions, wide disk-shaped lesions, or significant wedge-shaped abnormalities. The lesion's floor could be smooth, depressed, or acutely inclined [4].

Dentin exposure in NCCLs refers to the exposure of the tooth's dentin layer, which lies just beneath the enamel. Dentin is a porous and sensitive layer of the tooth that contains tiny tubes filled with fluid and nerve fibers. When NCCLs progress and reach the dentin layer, it can cause sensitivity to hot, cold, or sweet food and drinks, as well as pain and discomfort when chewing [5]. Dentin exposure can also lead to further decay, as the bacteria can easily penetrate the porous layer and reach the pulp, which can result in infection and potentially require root canal treatment. The most significant contributing element to dentin exposure above the gingival edge is NCCLs, which are also thought to be a risk factor for dentin hypersensitivity (DH). When the dentin is subjected to the oral environment and the dentin tubules are in close proximity to one another, DH, a painful condition, develops. Erosion has been regarded as the primary etiological reason for DH since it can open and enlarge the dentin tubules in addition to encouraging tooth structure loss. Depending on how abrasive the toothpaste is, brushing would thus serve as an adjuvant, accelerating the loss of dentin after erosion and also opening dentinal tubules [6].

Treatment for dentin exposure in NCCLs typically involves placing a restorative material such as composite resin or a glass ionomer cement to protect the exposed dentin and prevent further decay. However, it is found that these materials are not able to bond to the dentin, and therefore, substances like sodium fluoride and NovaMin are used in dentin remineralization [7,8]. In some cases, a dental crown may also be recommended to provide additional protection and support to the tooth [9]. It is important to address dentin exposure in NCCLs promptly to prevent further damage and preserve the affected tooth [10].

Examining teeth affected with NCCLs at the nanoscale level will help us know the morphology and help in making appropriate treatment plans. Furthermore, an energy-dispersive spectroscopy (EDS) study will allow us to know the presence of different elements present in the affected tooth and give us better knowledge about the dentin and its tubules. A similar study showed the nanoscale structures of dentin in non-carious cervical lesions [11]. Teeth were categorized into various categories like shallow, concave, wedge shaped, notched, and irregular non-carious cervical lesions. Using scanning electron microscopy (SEM), the morphological forms of the samples of extracted permanent anterior teeth of humans were examined. To our knowledge, there is no study that has tried to identify the elemental composition of these teeth affected with NCCLs. Therefore, this study aims to assess the nanoscale configuration of dentin in non-carious cervical lesions using SEM and color EDS.

## Materials and methods

**Figure 1:**
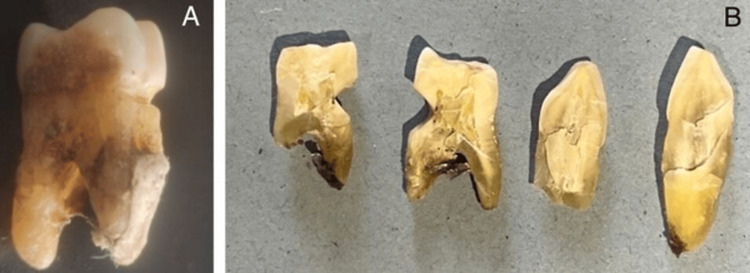
(A) Tooth showing non-carious cervical lesions. (B) Tooth samples sliced using a diamond bur

In this study, all the teeth used were derived from the extracted tooth repository of Saveetha Dental College and Hospitals, Chennai. These teeth were extracted in the early 2023. Two teeth with non-carious cervical lesions were manually assorted and selected for the study. At the time of the research, the teeth were in the dry form. Teeth were sterilized and stored in formalin before using them in further in the study. Teeth were then carefully sliced into 1-2 mm thickness transversely and longitudinally using a diamond burr in such a way that the non-carious cervical lesion was predominantly seen. To dehydrate, the teeth were kept in a hot air oven for about four hours at 70°C (Figures 1A-1B).

These tooth slices were then physically analyzed using a field emission scanning electron microscope (JSM-IT800 NANO SEM; JEOL Ltd., Tokyo) at a range of magnifications. Using SEM, a visual examination of a surface can help determine contaminants or unidentified particles, the root of a failure, and interactions between different types of materials [9]. SEM analysis is used for particle characterization in addition to surface evaluation. For example, wear debris produced during mechanical wear testing is characterized using SEM analysis. SEM analysis facilitates the assessment of the quantity, and morphological features of microscopic particles, enabling clients to comprehend the wear qualities of the material. This is done using high-magnification, high-resolution photography [11,12].

Analytical methods that enable the chemical characterization and elemental analysis of materials include energy-dispersive X-ray spectroscopy color-coded elemental mapping. When a substance is stimulated by an energy source, such as the electron beam from an electron microscope, some of the energy that has been absorbed can be released. The subsequent release of the energy difference results in the creation of an X-ray with a specific spectrum that depends on the atom it came from. This happens when an outer-shell electron with a greater energy steps in to take its place. This makes it possible to do a compositional analysis on a volumetric sample that has been stimulated by an energy source. The identification of an element is done by the position of the peaks in the spectrum, and the element's concentration is represented by the signal's strength. Chemically analyzed using energy-dispersive X-ray spectroscopy, various elements present in different compositions were analyzed.

## Results

**Figure 2:**
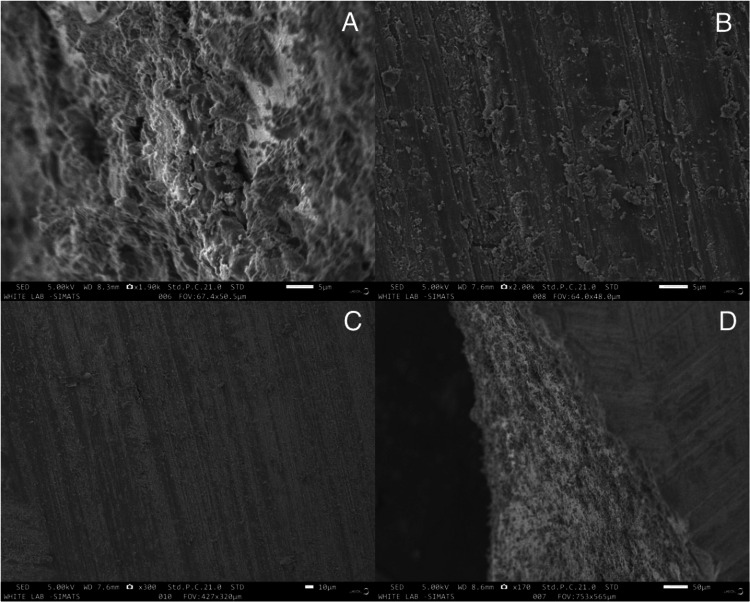
Dentinal tubules under a scanning electron microscope with different magnifications of (A, B) 5, (C) 10, and (D) 50 micrometers

The nanoscale surface morphology was viewed under SEM with various magnifications of 5, 10 and 50 micrometers, which is depicted in Figure 2. These SEM images show the beautiful arrangement of dentinal tubules in the exposed area.

**Figure 3:**
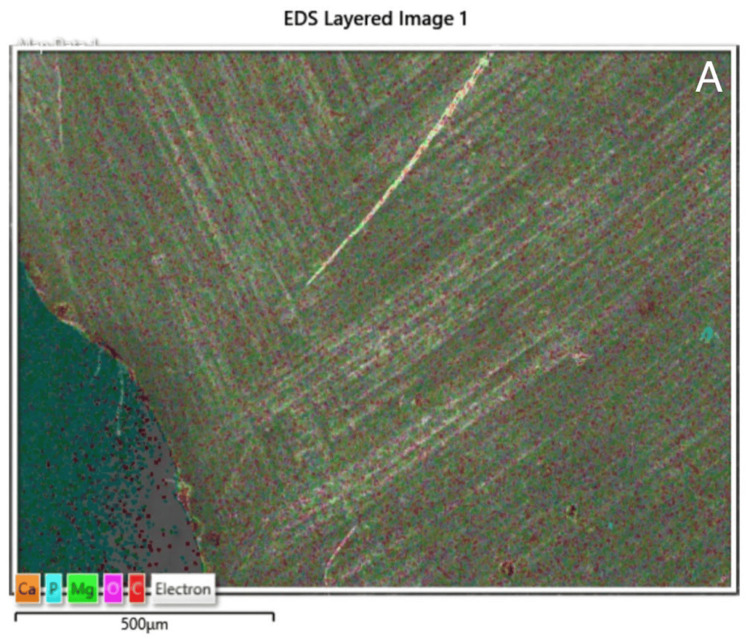
EDS color mapping of exposed dentinal tubules of teeth with NCCLs, under the magnification 500 micrometers EDS, energy-dispersive spectroscopy; NCCL, non-carious cervical lesion

**Figure 4:**
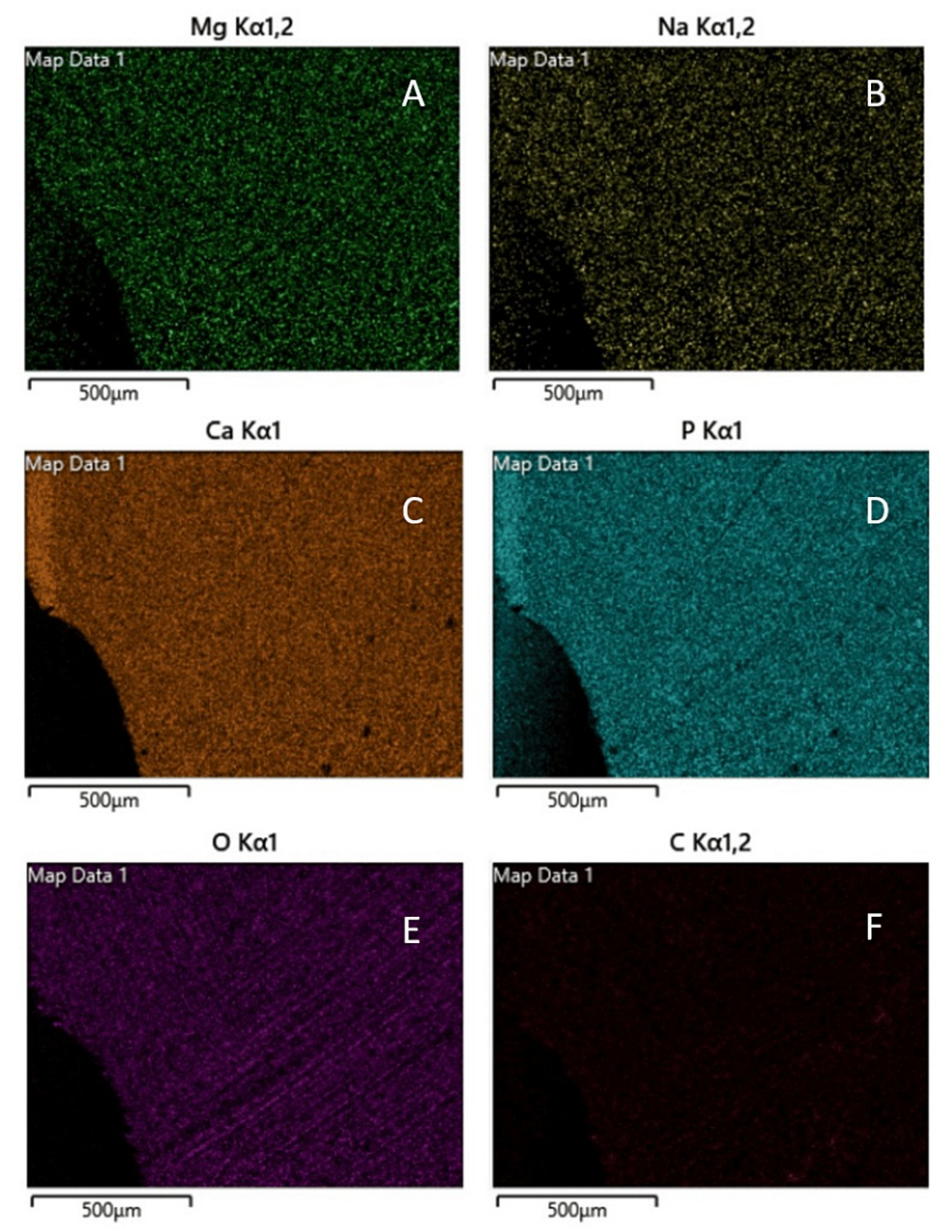
(A) Uniformly distributed magnesium atoms. (B) Uniformly distributed sodium atoms. (C) Uniformly spread calcium atoms. (D) Uniformly distributed phosphorus atoms. (E) Uniformly spread oxygen atoms. (F) Presence of carbon that is minimal and scattered

Coloured EDS image and elemental mapping of the tooth sample is shown in Figure 3. It showed the presence of various elements like calcium, phosphorus, magnesium, oxygen and carbon. All the elements were distributed uniformly on the tooth structure except for carbon that was very less in quantity and scattered (Figure 4).

**Figure 5:**
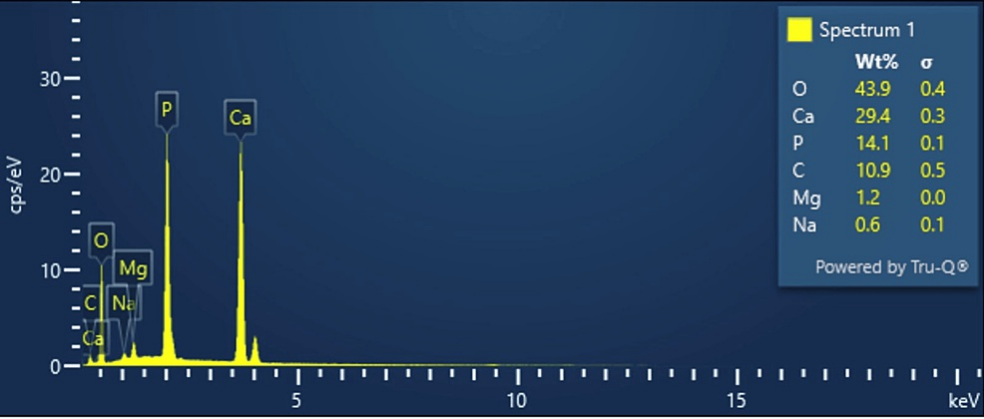
Energy-dispersive spectroscopy of the NCCL tooth sample NCCL, non-carious cervical lesion

The EDS graph of the tooth sample and the peaks show the quantity of the elements present in the sample affected with NCCLs (Figure 5). There were high peaks for phosphorus and calcium. Therefore, there were more counts per second per electron volt (cps/ev). The various elements present in the teeth with NCCLs varied with regard to concentrations (Table 1).

**Table 1:** Energy-dispersive spectroscopy showing weight percentages of various elements present in the study sample

S. no.	Elements	Weight %	% σ
1	O	43.9	0.4
2	Ca	29.4	0.3
3	P	14.1	0.1
4	C	10.9	0.5
5	Mg	1.2	0
6	Na	0.6	0.1

## Discussion

Dentinal tubule exposure describes the state in which the minuscule channels or tubules within the dentin layer of a tooth become visible. These tiny tubules connect the pulp chamber to the tooth's outer surface in the dentin, the layer that lies underneath the cementum and tooth enamel. The enamel or cementum covering of a healthy tooth shields the dentinal tubules from damage. Teeth can become sensitive and uncomfortable when the dentinal tubules are exposed [13]. Fluid and nerve endings are present in the tubules, and they may be triggered by outside elements like air, food that is sweet or acidic, or even changes in temperature [1]. The impacted tooth may become sensitive or painful as a result of this stimulation.

Dentin exposure in non-carious cervical lesions is a disorder marked by tooth structure loss in the cervical portion of the tooth, close to the gum line. NCCLs are frequently detected as shallow grooves, abrasions, or notches on the surface of teeth, and they frequently occur due to mechanical or chemical reasons rather than a dental disease, causing gingival recession leading to dentin exposure and hypersensitivity. In a study by Yoshizaki et al. in 2017, an interview was conducted to gather biographical data and identify any potential risk factors for DH and NCCLs [14]. A clinical examination was done to document the NCCLs, lesion form, and several occlusal variables. By using air and probe testing, DH was identified. Prevalence ratios and multilevel Poisson regression were used to evaluate the data, and the corresponding 95% confidence intervals were generated. The factors associated with NCCLs were aging, presence of premature contacts in maximum intercuspation and frequent consumption of wine and alcoholic beverages affecting the non-working side. DH was affected by aging, presence of NCCLs, premature contacts and frequent consumption of erosive and acidic fruit juices. In 2014, Sadaf and Ahmad did a cross-sectional study, and examinations were done to check for parafunctional habits, non-carious cervical lesions, damaged restorations, cracked cusps, occlusal facets, and brushing behaviors [15]. The development of NCCLs may come from an interplay of multiple elements, and it was discovered that occlusal pressures and occlusal trauma were not clinically demonstrated to be the primary factor in their creation. Wear on the cervical teeth is strongly correlated with overbrushing and using hard toothbrushes. Patients must be taught how to wash their teeth properly. Also, dentin demineralisation was found in majority of NCCL cases of the population. It can also be attributed that attrition of the occlusal surface and loss of dentin minerals were closely associated with larger and severe NCCLs. Also, these factors were reported to be the potential etiological factors for initiation and progression of these lesions, by Wada et al. [16]. Langenbach et al. performed a cross-sectional study and looked at the prevalence of NCCLs and their relationship with dentin hypersensitivity. To find DH, a probe and an air test were used. The hydrodynamic hypothesis is regarded as the current pain mechanism of DH. The teeth and periodontium's features, the oral environment, and outside factors all have an impact on the beginning and advancement of DH. There are several risk variables, many of which interact and always depend on personal sensitivity [17]. A similar study conducted by Soares et al. in 2021 found that DH and NCCLs are common conditions occurring in adults [18]. DH can also have a greater impact on oral health. Although DH is related to physical pain dimensions regardless of NCCLs, a higher impact was observed in cases with both NCCLs and DH.

Another study showed various types of surface structures and morphologies of NCCLs using SEM. Classification was done on 13 various types of features such as cracked, smooth, stippled, furrows, etc. [11]. This study did not find elemental compositions of the NCCLs. Therefore, this research fulfills the deficits of the previous study by analyzing the various elements present in NCCLs.

The small sample size was a limitation of this study. Further studies can be conducted on a variety of teeth by classifying them on the basis of morphology or location. The etiology of these specimens can be used for various other qualificative features. Novel treatment plans could be found in treating non-carious cervical lesions [13]. Such methods would have significant diagnostic implications for the therapeutic management of these diseases and would allow for the exploration of more conclusive correlations between the macroscopic anatomy and the qualitative aspects of the lesions, thereby establishing their etiology [19,20].

## Conclusions

We assessed the nanoscale configuration of dentin in non-carious cervical lesions using SEM and color EDS. Elemental mapping was also done to locate the presence of elements and the results showed that these elements were uniformly spread. Evidence indicates that NCCLs can be attributed as a predominant factor leading to gingival recession causing dentin exposure and hypersensitivity. NCCLs can also threaten the integrity of dentin; therefore, early diagnosis of non-carious cervical lesions is crucial to ensure proper treatment plans and therapeutic regimens. Further studies using advanced technologies are required to help us derive better treatment plans.
